# Competing endogenous RNAs (ceRNAs): new entrants to the intricacies of gene regulation

**DOI:** 10.3389/fgene.2014.00008

**Published:** 2014-01-30

**Authors:** Reena V. Kartha, Subbaya Subramanian

**Affiliations:** ^1^Center for Orphan Drug Research, Department of Experimental and Clinical Pharmacology, University of MinnesotaMinneapolis, MN, USA; ^2^Division of Basic and Translational Research Institute, Department of Surgery, University of MinnesotaMinneapolis, MN, USA; ^3^Masonic Cancer Center, University of MinnesotaMinneapolis, MN, USA

**Keywords:** competing endogenous RNAs, miRNAs ceRNAs, microRNAs, MREs, sponge effect, RNA-RNA crosstalk

## Abstract

The discovery of microRNAs (miRNAs) has led to a paradigm shift in our basic understanding of gene regulation. Competing endogenous RNAs (ceRNAs) are the recent entrants adding to the complexities of miRNA mediated gene regulation. ceRNAs are RNAs that share miRNA recognition elements (MREs) thereby regulating each other. It is apparent that miRNAs act as rheostats that fine-tune gene expression and maintain the functional balance of various gene networks. Thus MREs in coding and non-coding transcripts have evolved to become the crosstalk hubs of gene interactions, affecting the expression levels and activities of different ceRNAs. Decoding the crosstalk between MREs mediated by ceRNAs is critical to delineate the intricacies in gene regulation, and we have just begun to unravel this complexity.

## Introduction

In recent years, advances in genomic sequencing have led to several discoveries that can be considered exceptions to the central dogma of molecular biology. Much of the DNA that does not encode proteins has been shown to code for various types of functional RNAs that have important regulatory roles. The complexities in gene regulation increased logarithmically following the identification of microRNAs (miRNAs). miRNAs are 18–22 nucleotide long, evolutionarily conserved single-stranded RNA molecules that play a significant role in maintaining cellular identity and homeostasis (Bartel, 2004). miRNA mediated regulatory networks were identified as drivers in diverse disease conditions including cancers, where they function either as oncogenes or as tumor suppressors (Hofacker, 2007; Kapsimali et al., 2007; Moffett and Novina, 2007; Croce, 2009). This was followed by the discovery of several non-coding RNAs with regulatory functions in disease conditions (Rinn et al., 2007; Clark and Mattick, 2011; Sun et al., 2013). Several studies have demonstrated the complex mechanisms by which miRNAs regulate gene expression by interacting with multiple networks (Hammond, 2007; Yang et al., 2007; Sarver et al., 2010). This is because a single miRNA can regulate tens or hundreds of mRNA targets and, conversely, several miRNAs can regulate a single mRNA (Eulalio et al., 2008; Bartel, 2009; Friedman et al., 2009; Ebert and Sharp, 2012). Depending on the degree of homology of the seed sequence (6–8 nucleotide long miRNA region that determines target specificity), miRNAs can either completely degrade the target mRNAs (perfect base pairing) or act as road-blocks to translation (imperfect base pairing) (Yekta et al., 2004; Eulalio et al., 2008; Bartel, 2009). The multifaceted role of miRNAs can be appreciated by the recently described decoy activity of miRNAs, signaling a paradigm shift. Perrotti's group observed that in leukemic blasts, miR-328 is downregulated in a BCR/ABL-dependent manner (Eiring et al., 2010). In these cells, there is increased expression of the RNA binding protein hnRNP E2, which interacts with mRNA for a transcription factor *CEBPA*, inhibiting its translation. miR-328 functions as a decoy by competing with CEBPA mRNA for the hnRNP E2 binding site, thereby preventing the translational inhibition of CEBPA mRNA. This function is in addition to its canonical function of suppressing PIM1 protein kinase translation, but nonetheless is critical to the regulatory control of the blast cell oncogenesis.

**Figure 1:**
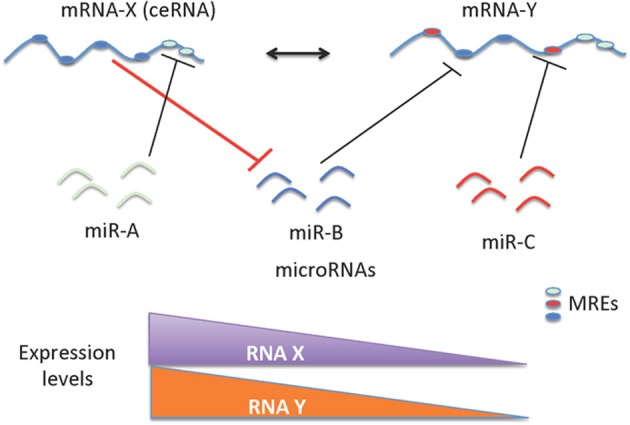
**Schematic representation of ceRNAs mediated gene regulation**. The regulation of miRNAs and mRNAs are mutual; as a consequence, levels of mRNA(s) containing relevant MREs could affect the levels and activity of another mRNA. The cross talk between mRNAs mediated by the MREs depends on the number and frequency of specific type of MREs. This cross talk is significantly influenced by the number and frequency of relevant MREs in the transcriptome of a given cell. The ceRNA-miRNA-mRNA cross talk maintains the overall activity and functional balance of gene networks in a cell. The ceRNA mediated regulation of mRNA-Y is illustrated here. mRNA-Y is a functional target of miR-B. However, the expression levels of mRNA-X that contains MREs for miRNA-B can act like a sponge to sequester the effect of miR-B on mRNA-Y (indicated with bold red line). Hence a decrease in expression levels of mRNA-X can also decrease the levels of mRNA-Y.

Gene expression can be regulated in *cis* by enhancer sequences or in *trans* by genes that encode transcription factors (that act as activators or repressors) or RNA binding proteins. Recently, several studies have demonstrated that both coding and non-coding RNA molecules can regulate gene expression in *trans* by acting as sponges of miRNAs (Karreth et al., 2011; Salmena et al., 2011; Tay et al., 2011; Karreth and Pandolfi, 2013; Su et al., 2013). Together these RNA molecules are called “competing endogenous RNAs” (ceRNAs), which constitute a major proportion of gene regulators. The identification of this level of gene regulation can explain the correlation between genome size and increase in species complexities. Although the number of protein-coding genes is comparable between lower organisms and humans, there is a proportional increase between the number of non-coding transcripts and the complexity among species (Mattick, 2011a,b). These ceRNAs share sequences recognized by the miRNAs called microRNA recognition elements (MREs). The ratio of noncoding to coding sequences in humans is about 47:1 (Frith et al., 2005) with up to 97% of the human genome constituting non protein-coding DNA (Tisseur et al., 2011). Further discovery of this additional layer of complexity in gene regulatory networks opens up new avenues for targeting therapy. The ceRNA concept, which describes the cross talk between RNA molecules mediated by miRNA recognition elements (MREs), is illustrated in Figure 1.

### miRNA sponges: a historical perspective

miRNAs regulate their target genes by binding to complementary sequences in the 3′ UTR. In order to understand the function of these miRNAs in biological systems, a general approach is to design miRNA sponges or target mimics that harbor several binding sites for a miRNA of interest. This concept of competitive target inhibition of miRNAs within a cell was first demonstrated by Phil Sharp's laboratory (Ebert et al., 2007). By this approach, one can decrease the number of miRNAs that are available to interact with an mRNA of interest, thus generating a loss-of-function phenotype in cell culture. At the same time, miRNA sponges were also observed to occur naturally.

The first endogenous miRNA sponge was discovered in plants and was found to moderate a miRNA mediated response to environmental stress (Franco-Zorrilla et al., 2007). The identified sponge was a non-coding RNA, Induced by Phosphate Starvation 1 (*IPS1*) with a binding site for miR-399, a phosphate starvation-induced miRNA. However, miR-399 binding does not induce degradation of the *IPS1* transcript due to mismatched nucleotides in the binding site, but rather results in sequestration of miR-399 from other target transcripts. Thus, *IPS1* can effectively function as a sponge inhibiting the number of miR-399 molecules available for regulating its target *PHO2* mRNA. *The Arabidopsis thaliana pho2* mutant is a phosphate over-accumulator. This mutant carries a mutation in the PHO2 gene, encoding a ubiquitin-conjugating enzyme (UBC24), that leads to a reduction in full-length transcripts. (Aung et al., 2006; Lin et al., 2008). This mutation causes symptoms related to inorganic phosphate (Pi) toxicity, as a consequence of enhanced Pi uptake, facilitated translocation of Pi from roots to shoots and impaired Pi remobilization from old to your leaves (Bari et al., 2006; Chitwood and Timmermans, 2007). Initially during phosphate starvation, *PHO2* is downregulated (Bari et al., 2006; Chitwood and Timmermans, 2007). In response to phosphate starvation, *IPS1* RNAs are induced. The latter can sequester miR-399 resulting in stabilization and increased accumulation of *PHO2* and, concomitantly, in reduced shoot phosphate content (Franco-Zorrilla et al., 2007). The authors coined the term “*target mimicry*” to define this mechanism of inhibition of miRNA activity.

This idea of target mimicry demonstrates unanticipated complexity in the network of RNA regulatory interactions and was the first natural example of self-regulation by RNAs. Subsequently, target mimicry has been exploited to determine the function of plant miRNA families using a high-throughput approach to generate a large-scale collection of knockdowns for *Arabidopsis thaliana* miRNA families (Todesco et al., 2010). This idea was followed by the discovery of several mRNAs that can competitively inhibit regulatory small RNAs in prokaryotes (Figueroa-Bossi et al., 2009; Mandin and Gottesman, 2009; Overgaard et al., 2009). In marmoset T cells transformed with primate virus Herpesvirus saimiri (HSV), downregulation of host miRNAs miR-16, -27, and -142-3p by viral non-coding transcripts called HSURs (H.saimiri U-rich RNAs) was observed (Cazalla et al., 2010). Additionally, protein expression of the miR-27 target gene *FOXO1* was observed to be upregulated in the presence of *HSUR1*, although neither the cellular compartment in which this interaction between HSUR and miRNA occurs nor the mechanism by which HSUR induces miRNA regulation are understood (Ebert and Sharp, 2010). Recently, sponge activity of viral RNA was confirmed by infection of mouse cells with murine cytomegalovirus resulting in rapid downregulation of anti-viral cellular miR-27 (Libri et al., 2012; Marcinowski et al., 2012). The authors in this study speculate that RNA mediated miRNA degradation could be a more general strategy employed by viruses to manipulate host cells. Another example for miRNA sponge activity in cancer progression is that of a non-coding RNA called “highly upregulated in liver cancer” (HULC), one of the most up regulated of all genes in hepatocellular carcinoma (Panzitt et al., 2007). Subsequently, Wang et al. documented that CREB (cAMP reponse element binding) protein is involved in upregulation of HULC and that HULC sequesters several miRNAs including miR-372, leading to derepression of its target protein, PRKACB, which in turn induces phosphorylation and activation of CREB (Wang et al., 2010). Thus *HULC* is an autoregulatory long non-coding RNA.

In spite of these observations of the occurrence of competitive inhibition, it was not until 2009 that a wider biological function was proposed for this regulatory mechanism. Following the discovery of natural miRNA sponges it was speculated that these regulatory RNAs exist to maintain periodicity in gene expression, by allowing brief spurts of miRNA activity followed by a pause during which time target mRNA levels can recover (Chitwood and Timmermans, 2007). Further, Seitz observed three paradoxes with respect to miRNA mediated gene regulation: (1) miRNA regulates several gene targets, yet the repression of only a few target genes significantly affects physiological function; (2) the extent of miRNA-mediated repression is remarkably lower as compared to endogenous genetic variations, which are well-tolerated; and (3) miRNA gene targets are significantly conserved among related species, but vary greatly between more distant species (Seitz, 2009). Seitz proposed that several of the computationally identified targets of miRNA can function as its competitive inhibitor, sequestering that miRNA, thereby preventing it from binding to its authentic mRNA target (Seitz, 2009). Seitz study postulates that miRNAs bind not only to their legitimate targets, whose activity is sensitive to small reduction in protein expression, but also to its pseudotargets that, on the contrary, are insensitive to such small reductions that can buffer and dilute miRNA activity. This formed the basis for the ceRNA hypothesis (Salmena et al., 2011), which was substantiated by the identification and characterization of several novel categories of ceRNAs in mammalian tissues. In the following section, we describe the various types of ceRNAs and provide a review of the experimental and/or theoretical evidences for their regulatory function. These studies demonstrate the competition between RNA molecules containing same MREs, leading to the sharp, rapid, and dynamic upregulation of target mRNAs.

### Protein coding RNAs as ceRNAs

A flurry of recent studies in mammalian cells and tissues demonstrated the competition between protein coding targets of miRNAs (Cesana et al., 2011; Karreth et al., 2011; Sumazin et al., 2011; Tay et al., 2011). These studies support a regulatory role for mRNAs independent of their protein coding function. Tay et al. tested the hypothesis that RNAs actively regulate each other through competitive sequestration of miRNA binding (Tay et al., 2011). The authors identified and validated several endogenous protein-coding transcripts that regulate phosphatase and tensin homolog (*PTEN*), using a combined computational and experimental approach. *PTEN* is a tumor suppressor gene, mutations in which have been implicated in several cancers. Further subtle changes in *PTEN* dose can dictate critical outcomes with respect to tumor initiation and progression (Trotman et al., 2003; Alimonti et al., 2010; Berger et al., 2011). Thus identifying ceRNAs for *PTEN* is of functional significance. This combinatorial analysis identified over 100 protein coding mRNAs that share MREs with *PTEN*. Furthermore, the authors observed the competing transcripts such as *CNOT6L* and *VAPA* to antagonize PI3K/AKT signaling, thereby contributing to growth- and tumor-suppressive properties. One additional *PTEN* ceRNA that has been functionally validated is Zinc finger E-box-binding homeobox 2 (ZEB2) (Karreth et al., 2011). Using *Sleeping Beauty* insertional mutagenesis screen in a mouse model of melanoma, the authors identified 33 putative *PTEN* ceRNAs. Further validation was performed using RNAi-mediated gene silencing in human melanoma cells. In this manner, silencing of 6 ceRNAs, including ZEB2, showed significant attenuation in PTEN levels in a miRNA-dependent, protein coding-independent manner. In addition to the regulatory function of the *ZEB2* transcript, its protein function is well established as a transcription factor. The ZEB2 transcription factor is an activator of epithelial-to-mesenchymal transition (EMT) and thus it is oncogenic and involved in the progression of epithelial cancers (Vandewalle et al., 2005; Gregory et al., 2008; Vandewalle et al., 2009). On the other hand, in this study the authors show that reduction of ZEB2 mRNA expression activates the PI3K/AKT pathway and enhances cell transformation. Reduction of ZEB2 mRNA expression commonly occurs in human melanomas and other cancers expressing low PTEN levels. Thus the mRNA and protein of ZEB2 have contradictory tumor suppressive and oncogenic functions, respectively. However, in light of a recent study showing that ZEB2 protein is oncosuppressive in melanoma (Caramel et al., 2013), the functional role of ZEB2 is context and tumor type dependent.

**Figure 2:**
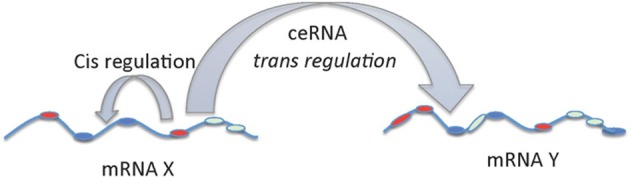
***cis* and *trans* regulation of mRNA**. The MREs in the 3′UTR of mRNA can act as *cis* regulatory elements affecting its own stability and levels via miRNAs. On the other hand, MREs in the mRNAs functioning as ceRNAs can regulate the levels and activity of another mRNA by *trans* regulation.

In a third study, large scale analysis of gene expression data with matched miRNA expression profiles in human glioblastomas identified ~7000 genes that can act as miRNA sponges or target decoys (Sumazin et al., 2011). Further biochemical analyses in cell lines demonstrated an intricate network regulating established driver genes including PTEN, PDGFRA, RB1, VEGFA, STAT3, and RUNX1, that allows cross talk between several canonical oncogenic pathways by titrating the activity of common miRNAs. Thus, although individual miRNAs contribute to small changes in gene expression, the combinatorial effect of multiple ceRNAs that interact via common MREs can be substantial and can explain the gene regulatory process in situations where there are no direct transcriptional or post-transcriptional interactions. While, MREs in a given transcript can regulate their own levels (*cis* regulation at transcript level) it can be noted that these MREs can also influence the levels and activity of other mRNAs (*trans* regulation) by competing for the miRNAs. mRNA mediated *cis* and *trans* regulation is schematically represented in Figure 2.

### Pseudogenes as ceRNAs

The identification of a novel biological role for the expressed pseudogene of PTEN was the first experimental proof for the cross talk between coding and non-coding RNAs (Poliseno et al., 2010). Poliseno et al. showed that in addition to protein-coding function, RNAs possess regulatory function and demonstrated this using mRNAs produced by the *PTEN* tumor suppressor gene and its pseudogene *PTENP1* (also known as PTENpg1, PTEN2, PTENψ1). They showed that MREs are conserved between *PTEN* and *PTENP1* and, as a consequence, overexpression of *PTENP1* 3′UTR correspondingly increased cellular levels of PTEN by sequestering all miRNAs that inhibit PTEN. This in turn led to growth inhibition. The authors also observed a direct correlation between the expression of *PTEN* and *PTENP1* in normal human tissues and prostate tumor samples suggesting these to be co-regulated. Further there was a direct relationship between *PTENP1* copy number and *PTEN* expression in colon cancer tissues, indicating that *PTENP1* transcript levels can regulate *PTEN* expression and thus act as a tumor suppressor (Poliseno et al., 2010). A similar observation was also reported in this study for KRAS gene: *KRAS1P* pseudogene pairs. The transcript levels of *KRAS* and *KRAS1P* were positively correlated in prostate cancer tissues. Interestingly, amplification of the KRAS1P locus is reported for several human tumors, including neuroblastoma, retinoblastoma, and hepatocellular carcinoma (Plantaz et al., 1997; Zimonjic et al., 1999; Van Der Wal et al., 2003). These observations indicate a proto-oncogenic role for KRAS1P.

Supporting the regulatory function of pseudogenes, Poliseno et al. also reported conservation in the MREs of specific miRNAs between a gene and its pseudogene, in the majority of the genes analyzed. For instance, *OCT4* pseudogenes OCT4-pg1, 3, 4, and 5 maintained validated binding sites for miR-145, *CDK4* pseudogene *CDK4PS* for miR-34 family, *FOXO3* pseudogene FOXO3B for miR-182, *KRAS* pseudogene *KRAS1P* maintained MREs for miR-143 and let-7 family, while *E2F3* pseudogene *E2F3P1* maintained validated binding sites for miR-17 family but not for miR-34 family. DNMT3A pseudogene DNMT3AP1 did not maintain MREs for miR-29 family and miR-143. Thus it seems that the function of a pseudogene generally mirrors its kindred gene function and contributes to a miRNA decoy mechanism (Poliseno et al., 2010). Pseudogenes can possess many of the same MREs that are harbored on the ancestral genes and thus act as perfect sponges (Salmena et al., 2011). It is noteworthy that many ribosomal genes have several pseudogenes that are differentially regulated (Balasubramanian et al., 2009), indicating intricately dynamic mechanisms for RNA cross talk.

Recently Johnsson et al. reported a hitherto unknown mechanism of regulation of *PTEN* transcription and translation by pseudogenes (Johnsson et al., 2013). The authors identified and characterized two antisense RNA (asRNA) isoforms, α and β which are encoded by pseudogene, PTENP1, in addition to the sense transcript that was shown to act as a miRNA sponge. The asRNA was generally expressed at higher copy numbers as compared to their sense counterpart (Johnsson et al., 2013). The α isoform of asRNA, acting in *trans* was shown to negatively regulate PTEN transcription by recruiting repressive chromatin remodelers, DNA methyltransferase 3A (DNMT3a) and Enhancer of Zeste (EZH2) to PTEN promoter region and catalyzes the formation of H3K27me3 chromatin mark (Johnsson et al., 2013). The isoform β, on the other hand, regulated PTEN at the post-transcriptional level. The β asRNA isoform interacted with the PTENP1sense transcript in the cytoplasm maintaining its stability, thereby affecting the sense miRNA sponge activity. This study is an elegant example of the intricate regulatory network involving pseudogenes and coding RNAs.

Another pseudogene acting as a miRNA sponge is OCT4-pg4, which is abnormally activated in hepatocellular carcinoma (Wang et al., 2013a). OCT4 is a POU transcription factor which is a pleiotropic regulator of gene expression in embryonic stem cells. The authors demonstrate that this pseudogene can protect OCT4 transcripts from miR-145 inhibition, thereby upregulating its protein expression. Further a significant correlation was observed between high OCT4-pg4 levels and poor prognosis, thus indicating this pseudogene to exert an oncogenic role in hepatocellular carcinoma.

### Long noncoding RNAs (lncRNAs) as ceRNAs

Any expressed RNA that is more than 200 nt in length and lacks obvious protein coding capacity is considered a lncRNA (Ponting et al., 2009). The dysregulation of lncRNAs has increasingly been linked to many human diseases, including cancers (Shi et al., 2013). The important roles played by lncRNAs in brain development, neuron function and maintenance, and neurodegenerative diseases are also becoming increasingly evident (Wu et al., 2013). Specific lncRNAs function to regulate gene expression either through epigenetic mechanisms or by post-transcriptional events such as mRNA processing and degradation by interacting with splicing factors and 3′ UTR elements, respectively (Guttman et al., 2009; Khalil et al., 2009; Huarte et al., 2010). Examples of lncRNAs involved in epigenetic regulation include X-inactive specific transcript (*XIST*) and large intergenic noncoding RNAs (lincRNAs). Notably, recent global analysis of Argonaute (Ago) bound transcripts through high-throughput sequencing of RNAs isolated by crosslinking immunoprecipitation (HITS-CLIP) technique suggests miRNA mediated regulation of lncRNAs (Licatalosi et al., 2008; Chi et al., 2009).

The ability of lncRNAs to function as ceRNA was first demonstrated in muscle differentiation (Cesana et al., 2011). The authors identified a muscle-specific lncRNA, linc-MD1, which governs the time of muscle differentiation by acting as a ceRNA in mouse and human myoblasts. This lncRNA sequestered miRNAs, miR-133 and miR-135 that specifically target protein coding genes MAML1 and MEF2C, needed to activate the differentiation program. Recently lincRNAs have also been shown to function as a miRNA sponge in human embryonic stem cell self-renewal (Cheng and Lin, 2013). Wang et al. showed that linc-RoR (regulator of reprogramming) functions as a miRNA sponge to post-transcriptionally regulate mRNAs of the core transcription factors for maintaining embryonic stem cell pluripotency (Wang et al., 2013b). Linc-RoR competes with mRNAs for *OCT4, NANOG* and *SOX2* for miR-145 binding. Consistent with its sponge effect, linc-RoR copy number was observed to be much higher than that of miR-145 (>100 vs. 10–20 copies/cell) in self-renewing embryonic stem cells compared to differentiating embryonic stem cells (20 vs. >500 copies/cell). Thus the sponge effect of linc-RoR can disappear once the embryonic stem cells start to differentiate.

### Circular RNAs (circRNAs or ciRs) function as ceRNAs

Occasionally RNAs that are covalently linked at the ends to form circular molecules have been described in literature. circRNAs were discovered in plants and were shown to be derived from viroids (Sanger et al., 1976). In lower organisms, circRNAs stem from self-splicing introns of pre-ribosomal RNA (Grabowski et al., 1981). In animals, many circRNAs overlap with the coding genes and arise from head-to-tail splicing of one or more exons. The best-known circRNA in animals is the antisense to the mRNA transcribed from *SRY* (sex-determining region Y) locus and considered circular testis-determining RNA (Capel et al., 1993). Nevertheless, circRNAs were considered rare until recently when several groups demonstrated their widespread occurrence within transcriptomes (Salzman et al., 2012; Wu et al., 2012; Jeck et al., 2013). It was only very recently that a ceRNA function was attributed to this highly prevalent class of conserved regulatory RNA molecules (Hansen et al., 2013; Memczak et al., 2013).

Memczak et al. adopted a novel computational methodology to screen RNA-seq libraries and detected thousands of human, mouse and *C. elegans* circRNA candidates (Memczak et al., 2013). They observed sequence conservation in these circRNAs and found several of these molecules to be specifically expressed in a cell type or developmental stage. Thus the authors speculated these previously disregarded molecules to perform a biological function, possibly in mediating gene regulation. Their hypothesis was proved by the characterization of a human circRNA, antisense to the cerebellar degeneration-related protein 1 transcript (CDR1as) also known as ciRS-7 (Hansen et al., 2013; Memczak et al., 2013). This brain-enriched circRNA harbors >70 MREs for the ancient miR-7 and forms complexes with Argonaute (AGO) protein in a miR-7 dependent manner. CDR1as (ciRS-7) is co-expressed with miR-7 in the brain and is co-localized in the P-bodies indicating that miR-7 compartmentalizes this circRNA to this organelle (Hansen et al., 2013). Further using functional approaches, it was demonstrated that the main role of CDR1as ciRS-7 is to bind miR-7 in neuronal tissues and ectopic expression of CDR1 is associated with morphological defects in the midbrain, phenocopying miR-7 knock-down (Hansen et al., 2013; Memczak et al., 2013).

**Figure 3:**
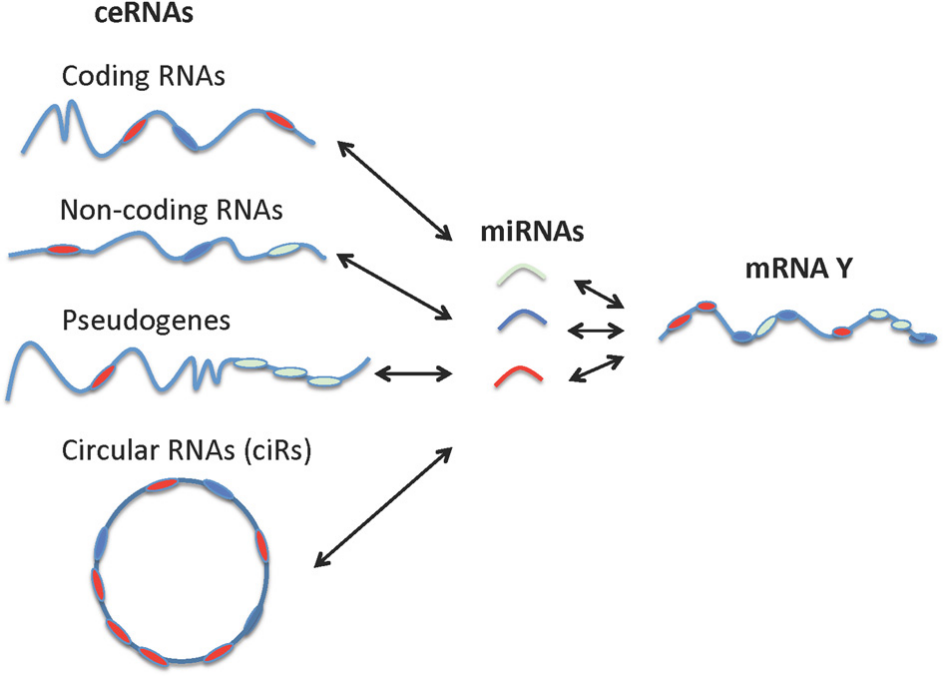
**Interactions between different types of ceRNAs, miRNA, and mRNA**. Coding RNAs and non-coding RNAs including pseudogenes, lncRNAs, and circular RNAs can function as ceRNAs.

It is noteworthy that non-coding RNAs, including pseudogenes, lncRNAs, and circRNAs behave more effectively as ceRNAs than do protein-coding RNAs since non-coding RNAs are not involved in the active protein translation process (Gu et al., 2009). While the miRNA sponges or miRNA-competing transcripts (protein-coding RNAs, pseudogenes, and lncRNAs) are expressed at low levels, contain a limited number of MREs, and are themselves subject to miRNA-mediated destabilization, circRNAs have been shown to be long-lived *in vivo* compared to their linear counterparts and are completely resistant to miRNA-mediated target destabilization (Hansen et al., 2013; Memczak et al., 2013). A recent study also showed that circRNAs can be used as “next generation” exogenous sponges that outperform linear ones (Liu et al., 2013a). Circular miRNA sponges against miR-21 or miR-221 obtained using the self-splicing permuted intron-exon sequences derived from T4 bacteriophage gene td (Liu et al., 2013b) were introduced into malignant melanoma cell lines and resulted in excellent anti-cancer effects. In sum, the non-coding RNAs vast outnumber the protein coding genes and are extremely versatile in their mechanism of action (Djebali et al., 2012; Guttman and Rinn, 2012). A schematic representation of various types of ceRNAs and their unifying role in tweaking mRNA expression level and activity is provided in Figure 3.

## Identification of ceRNAs

### Computational methods

The knowledge of precise numbers, types, and positions of MREs are very important for identification of ceRNAs. Although several target prediction algorithms are available, many are not successful in identifying all the important targets as the criteria for identifying targets are still evolving (Bartel, 2009). Tay et al. developed an approach termed mutually targeted MRE enrichment (MuTaME), involving integrated computational analysis and experimental validation (Tay et al., 2011). The authors used Rna22 miRNA target prediction algorithm (Miranda et al., 2006) to generate the MuTaME scores for the protein coding transcripts in humans. The MuTaME program evaluates a potential ceRNA based on (a) the number of miRNAs that are shared between transcripts of interest, (b) the number of predicted MREs in the transcript for a particular miRNA and the sequence span in which they are represented, (c) the distribution of the MREs of a particular miRNA on the transcript and (d) the relation between the total numbers of MREs predicted in a transcript to the total number of miRNAs that recognize these MREs. Using the MuTaME prediction algorithm Tay et al. (2011) demonstrated the physical association of PTEN-targeting miRNAs with *PTEN* ceRNA *CNOT6L*.

In order to assess the range and potential of miRNA-mediated regulatory interactions with a tumorigenic role, a novel multivariate analysis method called Hermes was developed (Sumazin et al., 2011). Hermes uses a large collection of mRNA and miRNA expression profiles obtained from same tumor samples to identify modulators of miRNA activity. Further, in order to predict the ceRNA network, the HERMES algorithm also considers non-coding RNAs. The program uses mutual and conditional mutual information to quantify the dependency of one variable over the other variable, which in this case is affected by potential ceRNAs. Using this program, the authors were able to predict the sponge interactions among ceRNAs in a glioblastoma dataset (Sumazin et al., 2011).

Our group subsequently developed a database named “competing endogenous RNA database” (ceRDB) that can help identify ceRNAs for a given mRNA. The program examines the co-occurrence of MREs in the mRNAs (3′ UTRs) on a genome-wide basis to predict ceRNAs for a specific mRNA targeted by miRNAs (Sarver and Subramanian, 2012). The ceRNA finder database is available through www.oncomir.umn.edu/cefinder. The program uses over 50,000 conserved human miRNA-mRNA target interactions obtained from Targetscan predictions with a matrix containing 153 miRNA families and 9448 target mRNAs. For each mRNA, the interactions are scored based on the sum of the total number of miRNA binding sites for a given miRNA. The interaction scores based on the MRE frequency is sorted to predict the top 50 potential ceRNAs for a gene of interest.

More recently a mathematical mass-action model to determine the optimal conditions for ceRNA activity *in vivo* was described by Pandolfi's group (Ala et al., 2013). The program mathematically models the complex network environment in which ceRNAs can function with optimal activity. Furthermore, the authors found that ceRNAs are responsive to modulations in the levels of various transcription factors. Thus, the deregulation of one ceRNA can have dramatic effect on the integrated ceRNA and transcriptional network. Besides the mass-action model there are also several other model systems reported (Bosia et al., 2013; De Giorgio et al., 2013; Figliuzzi et al., 2013; Noorbakhsh et al., 2013). Linc2GO, a lincRNA (long intergenic non-coding RNA) function annotation database was recently generated to predict lincRNA functions based on ceRNA hypothesis (Liu et al., 2013a).

The critical aspects of ceRNA identification are co-expression of the ceRNA, expression of miRNAs that commonly target the ceRNAs and the physical association (in part determined by the binding site availability due to RNA secondary structures) of miRNAs with the ceRNAs. Although various miRNA target prediction programs use algorithms to include seed sequence matches, evolutionary conservation of binding sites, and flanking secondary structures (Bartel, 2009), they predominantly focus on the 3′UTR regions to predict the physical association between miRNAs and target genes. Based on the recent findings that pseudogenes, lncRNAs and circular RNAs, can potentially function as ceRNAs, it becomes difficult to precisely determine the complete spectrum of all ceRNAs for a given gene of interest, unless non-coding RNAs are included in the target prediction algorithms.

### Experimental methods

Several biochemical techniques have also enabled the experimental identification and functional validation of ceRNAs. For instance, Argonaute high-throughput sequencing of RNAs isolated by crosslinking immunoprecipitation (Ago HITS-CLIP), photoactivatable-ribonucleoside-enhanced crosslinking and immunoprecipitation (PAR-CLIP) and RNA immunoprecipitation are some platforms that, by providing a footprint of all the miRNAs physically bound to a given RNA, can enable identification of MREs and will provide insights on ceRNA regulatory networks (Thomas et al., 2010).

## ceRNAs: what next?

It is becoming increasingly evident that regulation of gene expression through competition for miRNA binding is a general phenomenon. The understanding of gene regulation at this complexity has provided a functional explanation on how certain previously known genes with no direct interactions are inter-linked, as exemplified in the studies involving *PTEN*. But still, there is more to be unraveled and learned.

It is evident that the relative production and turnover rates of miRNAs, their target RNAs and ceRNAs can dictate the extent and duration of gene regulation (Arvey et al., 2010; Ala et al., 2013). There should be significant changes in ceRNA expression in order to relieve the miRNA repression of target mRNAs. Similarly, the magnitude of expression of the sequestered miRNAs should not be very low or abundant, as both conditions can obliterate the competition. Another factor that contributes to efficient competition is the number of miRNAs that a ceRNA can sponge. This in turn depends on the subcellular distribution and interaction of ceRNAs with RNA-binding proteins and ribosomes. Both ceRNAs and miRNAs should occur in the same tissue or cell type or cellular compartment at a specific time. Yet another factor that needs to be kept in mind is that not all MREs on ceRNAs are equal when it comes to binding a specific miRNA. This will depend on the nucleotide composition of MREs. All these details need to be clearly worked out to better appreciate the role of ceRNAs.

Another potential area for future research is in understanding the role of ceRNAs in disease pathophysiology. Several studies have analyzed gene or miRNA expression profiles in disease conditions. With the emergence of ceRNA mechanisms, what is warranted is a comprehensive analysis of both gene and miRNA expression in the same pathological sample. This will allow for identification of novel pathways that are deregulated in disease condition and decipher novel interactions between relevant signaling pathways. The bioinformatics tools that have been developed in recent years can contribute largely to this amount of global analyses.

Additionally, disease characterization in general involves extensive sequence analysis of coding regions to identify novel disease-causing mutations. These can be due to deletions, amplifications or chromosomal translocations as evident in cancer. With the emergence of ceRNAs, the search for perturbations should go beyond coding regions to include UTRs and introns as these can cause changes in MREs, which in turn can affect the complex regulatory circuits. For instance, gross genomic loss and amplification can have dramatic consequences for ceRNAs present in the region (Salmena et al., 2011). Similarly, chromosomal translocations can lead to UTR swaps (Stephens et al., 2009). Aberrant alternative splicing and alternative cleavage and polyadenylation events leading to shortening of 3′UTRs is another common mechanism in cancer (Mayr and Bartel, 2009).

In addition to massive genomic rearrangements, point mutations can lead to genetic disorders as observed with inborn errors of metabolism. It is important to note that even though point mutations abolish protein function, the mutant transcripts retain complete ceRNA function. Alternatively, point mutations in the MREs can abolish potential ceRNA functions, thereby perturbing the network. Sometimes these mutations are often overlooked, since they are not part of the functional protein coding sequence. Thus it is increasingly becoming important to have a whole genome approach when it comes to understanding disease. Often there is no correlation between the phenotype and genotype, suggesting genetic modifiers to contribute to the disease. With increasing evidence for miRNA mediated cross-talk, ceRNAs of a given disease-causing gene can potentially act as the modifier.

MRE mediated cross-talk between transcripts is expected to change our approaches in understanding disease mechanisms. For example, in microarray data analysis, traditionally only significant fold-changes are considered while interpreting gene expression patterns. Knowing that the number of MREs and its distribution can alter gene expression levels and its activity, even an insignificant fold-change in large number of genes is relevant and can considerably contribute a number of MREs that can affect the balance and dynamics of miRNA-mRNA interactions, which in turn affect cellular functions. Further, it is also possible that the transcripts with longer 3′UTR may function as “*master MRE*” containing genes and splice variants of these transcripts can also affect cell function. Gene regulation mediated by ceRNAs can also impact targeted therapy and therapeutic response. Since transcripts can independently function as ceRNAs, targeting their resulting proteins by small molecule inhibitors can in turn affect downstream the transcripts stability and levels that may contribute to undesirable side effects on treatment outcomes by the protein inhibitors. Modulation of levels of ceRNAs may be a strategic alternative to directly targeting a protein of interest. Further complicating this scenario is the recent finding that miRNAs can function as agents of intercellular communication (Arroyo et al., 2011; Lou et al., 2012; Steer and Subramanian, 2012). It is noteworthy that exosomes, which recently gained a lot of attention with regard to its ability to mediate intercellular communication (Mittelbrunn et al., 2011), may harbor both miRNAs and ceRNAs. This suggests that the miRNA pool in a cell is in constant flux, making it more challenging to understand gene regulation. Hence, it is critical to understand the spatial and temporal relationships between the relevant ceRNAs, mRNA, and miRNAs.

## Closing remarks

ceRNAs mediated gene regulation is an emerging area of investigation that will significantly increase our understanding of disease pathobiology, particularly cancer. In general, multiple genes and signaling pathways are deregulated in cancer. Many of these genes have a redundant function that obscures identification of therapeutically relevant target genes. In addition, contradictory functions of mRNAs (as ceRNA) and proteins of a gene further challenge the identification of relevant target genes. The *trans* regulations of ceRNAs add yet another layer of complexity to gene regulation that needs to be carefully investigated for key oncogenes and tumor suppressors implicated in various cancer types.

Overall, the normal functioning of cells depends on the maintenance of balance between complex regulatory systems. This is achieved by dynamic and fine adjustments and through the ability to rapidly return to normal threshold levels when imbalances are detected. If the system fails to balance beyond threshold levels, the cells manifest a disease condition. The challenges we face is in understanding how this balance is maintained in physiological conditions and conversely, how it is altered in disease. The recent ENCODE project results suggest that about 85% of the human genome is transcribed, the majority of which are lncRNAs (Djebali et al., 2012; Hangauer et al., 2013). With the recent discovery of many novel small and long non-coding RNA types with distinctive functions, we are just beginning a new chapter in gene regulation and disease biology with more “*unknown unknowns*.”

### Conflict of interest statement

The authors declare that the research was conducted in the absence of any commercial or financial relationships that could be construed as a potential conflict of interest.

## Abbreviations

ceRNA: competing endogenous RNAs
ciRs: circular RNAs
lncRNAs: long non-coding RNAs
miRNA: microRNA
MREs: microRNA recognition elements
UTRs: Untranslated region.
